# Incidence of post-traumatic endophthalmitis following repaired open globe injury: impact of prophylactic intracameral moxifloxacin

**DOI:** 10.1038/s41433-026-04338-y

**Published:** 2026-02-26

**Authors:** Karim Mohamed-Noriega, Alicia J. Hernández-Moreno, Fernando Morales-Wong, Jibran Mohamed-Noriega, Jesús H. González-Cortés, Gerardo Villarreal-Mendez, Jesús Mohamed-Hamsho

**Affiliations:** Ophthalmology Department, University Hospital and Faculty of Medicine, Autonomous University of Nuevo León (UANL), Monterrey, México

**Keywords:** Outcomes research, Infectious diseases, Public health

## Abstract

**Background/objectives:**

To assess the effect of intracameral moxifloxacin (ICM) after repaired open globe injury (OGI) in reducing the incidence of post-traumatic endophthalmitis.

**Subjects/methods:**

Ambispective, consecutive, comparative study of patients operated of OGI repair. Patients were divided into two groups: those without intracameral antibiotic prophylaxis (No-ICM) (operated between 2002 and 2012) and those with ICM prophylaxis at the end of the procedure (ICM) (operated between 2013 and 2023). Both received standard povidone-iodine antisepsis. Endophthalmitis was defined as severe intraocular inflammation associated with hypopyon and/or vitritis.

**Results:**

424 eyes (424 patients) were included, 294 in ICM group and 130 in No-ICM group. Male sex was 85.7% and 90.8% in ICM and No-ICM groups, respectively. The ICM group was significantly older than No-ICM group (median (IQR): 26 (14–41) vs. 21.5 (12–34), *p* = 0.025). The proportion of patients younger than 18 years was similar between ICM and No-ICM group (33.1% vs. 38.5%, *p* = 0.275). Post-traumatic endophthalmitis was significantly lower in ICM (2/294, 0.7%) compared to No-ICM (5/130, 3.9%) (*p* = 0.031) group. Sensitivity analysis age-adjusted confirmed the protective effect of ICM for endophthalmitis after OGI was independent of age (adjusted OR: 0.20; 95% CI: 0.04–0.86; *p* = 0.031). The relative risk of endophthalmitis with ICM compared with no-ICM was 0.18 (0.04–0.78), with relative risk reduction of 82%, absolute risk reduction of 3.2%, and a number needed to treat of 32 to prevent one case of endophthalmitis following OGI repair.

**Conclusions:**

ICM at the end of surgery reduces the incidence of post-traumatic endophthalmitis after OGI repair, compared with no intracameral antibiotic prophylaxis.

## Introduction

Postoperative endophthalmitis is a feared complication that can occur after any intraocular surgery [1, 2]. It refers to a severe inflammation associated with a bacterial or fungal infection within the eye, including the vitreous and/or aqueous humour [2–4]. Diagnosis is primarily clinical, with additional support from microbial culture of intraocular fluids [3]. A negative culture is observed in ~30% of cases; however, this does not rule out the diagnosis of endophthalmitis [3].

Endophthalmitis after cataract surgery has an approximate prevalence that ranges globally from 0.021% to 0.32% [5–8]. Povidone-iodine (PI) preparation of the eye and periocular skin has historically been the gold standard for endophthalmitis prophylaxis [9]. Intracameral, topical, subconjunctival, and oral antibiotics are other complementary alternatives [5, 9–11]. Currently, perioperative systemic antibiotics and preoperative topical antibiotics are not a standard of care and have not been shown to be superior to povidone-iodine alone [7, 12]. Intracameral antibiotic prophylaxis is widely accepted and has been investigated after cataract surgery, demonstrating a significant reduction of post-operative endophthalmitis [5, 7, 8, 10, 13–15]. The choice of prophylactic intracameral antibiotic at the end of intraocular surgical procedures varies internationally; when it is used, it is often cefuroxime, moxifloxacin, or vancomycin [5, 9, 12, 16]. Since 2013, in our tertiary referral centre in Monterrey, Mexico, intracameral moxifloxacin (ICM) has been routinely used as part of the prophylactic standard procedure at the end of intraocular surgeries such as cataract surgery and open globe injury (OGI) repair. ICM is preferred by many ophthalmologists because it is readily available, does not need refrigeration, does not need dilution, and is cost-effective [9, 10, 17–19]. Furthermore, a systematic review and meta-analysis by Kessel et al. provided high to moderate evidence of reduced risk for post-cataract endophthalmitis through intracameral use of cefuroxime, cefazolin, or moxifloxacin in comparison to povidone iodine alone [12].

Traumatic OGI frequently causes severe vision loss, bacterial endophthalmitis following surgical repair can significantly worsen visual outcomes in these cases [20]. There is limited evidence regarding the use of intracameral moxifloxacin as prophylaxis for endophthalmitis after repaired OGI. A systematic review by Thevi et. al. concluded that the use of intracameral or intravitreal antibiotics at the end of OGI repair should be considered to reduce the risk of endophthalmitis; however, no studies on ICM were included in this systematic review [21].

This study aims to assess the knowledge gap regarding whether ICM is more effective than no intracameral antibiotic prophylaxis in reducing the incidence of post-traumatic endophthalmitis after repaired OGI.

## Methods

Data were collected retrospectively from an ambispective cohort study of patients who underwent surgical repair of OGI at the ophthalmology department of the University Hospital and the Faculty of Medicine of the Autonomous University of Nuevo Leon, a tertiary care university hospital in Monterrey, Mexico, between 2002 and 2023. Retrospective data were collected from case files from 2002 to 2012, and prospective data from 2013 to 2023. The study received institutional ethics committee approval (registration number OF13-002) and was conducted following good clinical practices and the Declaration of Helsinki. All prospective patients (or their next of kin for paediatric patients) read and provided written informed consent to participate.

Patients of all ages and sexes undergoing OGI repair surgery at our institution were included. To analyse the role of the prophylactic ICM in preventing endophthalmitis, it was decided to eliminate from the study those with less than two weeks of follow-up as this short follow up might hinder the detection of endophthalmitis. Also, those who received intraocular antibiotics other than ICM prophylaxis were eliminated because any intraocular antibiotic used could obscure the actual effect of ICM in preventing endophthalmitis. Additionally, patients who underwent primary enucleation instead of OGI repair were eliminated, since the study focused only on patients with repaired OGI. Patients with a preoperative clinical diagnosis of endophthalmitis were also eliminated to avoid including individuals who already had intraocular infections at the time of OGI repair, which could obscure the benefit of the prophylactic antibiotic due to existing infection. Finally, all OGI repair surgeries performed elsewhere were excluded because it was not possible to determine if intraocular antibiotics were used or if there was preoperative endophthalmitis. Before 2013, in our institution, intraocular antibiotics were not routinely used. Since 2013, ICM has been used in a standardised way in all cases of OGI repair surgery. Patients were divided into two groups: those operated on between 2002 and 2012, where pre-operative antisepsis with PI (8% in the periocular area and 5% in the conjunctival sac) was applied for 5 min and did not receive intracameral antibiotic prophylaxis (No-ICM group). Those operated on between 2013 and 2023 received the same standard PI antisepsis previously mentioned plus ICM prophylaxis, where 0.1 mL of preservative-free moxifloxacin 0.5% (Vigamoxi®, Alcon, USA) was injected into the intracameral space at the end of the surgery (ICM group). In cases where the wound did not involve the anterior chamber, a standard peripheral corneal paracentesis was created, and intracameral moxifloxacin was administered through this incision at the end of the procedure, as previously described.

Patients underwent ophthalmologic evaluation at presentation, including best-corrected visual acuity (BCVA) or pinhole visual acuity, uncorrected visual acuity (UCVA), mechanism of lesion, wound length, and wound zone, ocular trauma score (OTS), anterior segment photograph, and when possible, B-scan ultrasound. X-rays and CT scans of the skull were also performed in situations where the injury mechanism suggests a possible intraocular or intraorbital foreign body. An ophthalmic examination was performed, and information regarding OGI was collected. Intraocular foreign body (IOFB), traumatic cataract, time intervals of accident-to-ophthalmic consultation, and accident-to-surgery were recorded in hours.

Patients received comprehensive care through a multidisciplinary approach, involving retina, cornea, anterior segment, and glaucoma clinics. All surgeries were performed by ophthalmologists experienced in OGI repair and were assisted by resident ophthalmologists in training. Intraoperative cultures of aqueous humour and wound edges were taken. After the surgery, each case was closely monitored during follow-up visits as needed. In the event of postoperative endophthalmitis, a standardised postoperative endophthalmitis report was completed (Supplementary Material 1). This report serves as an institutional audit of postoperative endophthalmitis and includes preoperative factors, surgical details, clinical presentation on the day of diagnosis, initial treatment, and visual acuity. Our operational definition of endophthalmitis for this study was a clinical finding of severe intraocular inflammation associated with either the presence of hypopyon and/or vitritis, irrespective of vitreous/aqueous cultures.

All the retrospective information was collected from the patient’s files, and all the prospective data were captured in a predesigned worksheet, then transferred to an excel database and later statistically analysed with IBM SPSS (IBM Corp. Released 2017. IMS SPSS Statistics for Windows, version 25.0. Armonk, NY: IBM Corp.). Continuous variables were evaluated for normality using Kolmogorov–Smirnov, Shapiro–Wilk tests, as well as visual inspection of histograms and Q-Q plots. The Mann–Whitney U Test was used to compare independent nonparametric data and was reported as median (interquartile range, IQR). Student’s *t* test was used to compare independent parametric data and was reported as mean ± standard deviation. Categorical variables were reported as counts and percentages. The Chi-square test was used to compare categorical data when expected event counts were greater than 5, and Fisher’s exact test was used when expected event counts were 5 or fewer. To quantify the strength of the observed relationship between the intervention ICM and the prevention of endophthalmitis after OGI repair, statistical analyses were performed using the epiR package (version 2.0.86) within the R Statistical Software environment (v4.4.3; R Core Team, 2025) [22, 23]. The Relative Risk (RR) and its 95% confidence interval (CI) were estimated using the Miettinen-Nurminen method. The Absolute Risk Reduction (ARR) and Number Needed to Treat (NNT), along with their 95% CIs, were derived directly from the RR estimates applied to the observed control group incidence. A sensitivity analysis using Firth’s penalised logistic regression was conducted to assess the protective effect of ICM adjusted for age using the logistf package (version 1.26.1) within the R Statistical Software environment (v4.4.3; R Core Team, 2025) [22, 24]. Statistical significance was considered when *p* value was <0.05.

## Results

Data of 441 patients who underwent OGI were collected. 13 patients were eliminated from the No-ICM group due to the use of intracameral antibiotics. Additionally, 4 patients with a preoperative diagnosis of endophthalmitis were eliminated (1 from the No-ICM group and 3 from the ICM group). Finally, a total of 424 eyes from 424 patients were included in the analysis, 130 in the No-ICM group and 294 in the ICM group.

Male patients were more frequently affected in both groups (85.7% and 90.8%, ICM and No-ICM group, respectively) with no significant difference between groups (*p* = 0.150). ICM group was significantly older than No-ICM group (median (IQR): 26 (14–41) vs. 21.5 (12–34), *p* = 0.025). The percentage of patients under 18 was 33.1% and 38.5% in ICM and No-ICM group, respectively (*p* = 0.275). Both groups have a similar proportion of patients with diabetes and hypertension (Table 1). The wound location is as follows: cornea (55.4% and 53.8%; *p* = 0.761), cornea-limbus (6.8% and 10.8%; *p* = 0.166), sclera (17.7% and 17.7%; *p* = 0.999) and corneo-sclera (20.1% and 17.7%; *p* = 0.568) in the ICM and No-ICM group, respectively. There was a significant difference in the mechanism of injury showing: stone (5.4% and 11.5%, ICM and No-ICM group, respectively; *p* = 0.026) and sharp metallic object (49.3% and 36.9%, ICM and No-ICM group, respectively; *p* = 0.018). Sharp metallic objects were more frequently seen in both groups. Zone 1 was more frequently affected in both groups (56.8% and 58.5%, ICM and No-ICM groups, respectively). Detailed demographic characteristics, preoperative variables, wound characteristics, and mechanisms of injury are presented in Table 1.

**Table 1:** Demographics in No-ICM group and ICM group.

Characteristics	All *N* = 424	No-ICM *N* = 130	ICM *N* = 294	*P*^a^
**Endophthalmitis**				
All patients	7 (1.7%)	5 (3.9%)	2 (0.7%)	**0.031**^b^
Only under 18 years old	4 / 147 (2.7%)	2 / 50 (4.0%)	2 / 97 (2.1%)	0.242
**Sex**				
Male	370 (87.2%)	118 (90.8%)	252 (85.7%)	
Female	54 (12.7%)	12 (9.2%)	42 (14.3%)	0.150
**Age**^a^				
Median (IQR)	24 (14-39)	21.5 (12-34)	26 (14-41)	**0.025**
Patients under 18 years old	147 (34.7%)	50 (38.5%)	97 (33.1%)	0.275
Type 2 Diabetes	10 (2.4%)	3 (2.3%)	7 (2.4%)	>0.999
Hypertension	11 (2.6%)	1 (0.8%)	10 (3.4%)	0.185
**Preoperative visual acuity**				
NLP	13 (3.1%)	5 (3.8%)	8 (2.7%)	0.549
LP	73 (17.2%)	22 (16.9%)	51 (17.3%)	0.915
HM	138 (32.5%)	38 (29.2%)	100 (34.0%)	0.332
CF	76 (17.9%)	25 (19.2%)	51 (17.3%)	0.641
20/400 or better	108 (25.5%)	29 (22.3%)	79 (26.9%)	0.320
Not evaluated	16 (3.8%)	11 (8.5%)	5 (1.7%)	**0.002**
**Mechanism of injury**				
Stone	31 (7.3%)	15 (11.5%)	16 (5.4%)	**0.026**
Glass	66 (15.6%)	25 (19.2%)	41 (13.9%)	0.166
Sharp metallic object	193 (45.5%)	48 (36.9%)	145 (49.3%)	**0.018**
Wooden stick	26 (6.1%)	7 (5.4%)	19 (6.5%)	0.670
Tree branch	42 (9.9%)	14 (10.8%)	28 (9.5%)	0.692
Firework	7 (1.7%)	3 (2.3%)	4 (1.4%)	0.443
Blunt object	3 (0.7%)	2 (1.5%)	1 (0.3%)	0.224
Punch	13 (3.1%)	4 (3.1%)	9 (3.1%)	>0.999
Others	43 (10.1%)	12 (9.2%)	31 (10.5%)	0.680
**Accident-repair interval**				
<24 h	155 (36.6%)	85 (65.4%)	70 (23.8%)	**<0.001**
24–72 h	159 (37.5%)	29 (22.3%)	130 (44.2%)	< **0.001**
>72 h	110 (25.9%)	16 (12.3%)	94 (32%)	< **0.001**
**Location**				
Cornea	233 (54.9%)	70 (53.8%)	163 (55.4%)	0.761
Cornea-limbus	34 (8.0%)	14 (10.8%)	20 (6.8%)	0.166
Sclera	75 (17.7%)	23 (17.7%)	52 (17.7%)	0.999
Cornea-sclera	82 (19.3%)	23 (17.7%)	59 (20.1%)	0.568
**Zone**				
Zone 1	243 (57.3%)	76 (58.5%)	167 (56.8%)	0.750
Zone 2	48 (11.3%)	6 (4.6%)	42 (14.3%)	**0.004**
Zone 3	10 (2.4%)	3 (2.3%)	7 (2.4%)	> 0.999
Zone 1 and 2	61 (14.4%)	19 (14.6%)	42 (14.3%)	0.929
Zone 2 and 3	22 (5.2%)	6 (4.6%)	16 (5.4%)	0.723
Zone 1, 2 and 3	40 (9.4%)	20 (15.4%)	20 (6.8%)	**0.005**
Intraocular foreign body	33 (7.8%)	4 (3.1%)	29 (9.9%)	**0.016**
**Transoperative steroid**				
Subconjunctival or periocular	49 (11.6%)	20 (15.4%)	29 (9.9%)	0.101
Intraocular	2 (0.5%)	1 (0.8%)	1 (0.3%)	0.520
Intravenous	5 (1.2%)	3 (2.3%)	5 (0.7%)	0.171
No steroid	368 (86.8%)	106 (81.5%)	262 (89.1%)	**0.034**
**Postoperative visual acuity (2 weeks)**				
NLP	21 (4.9%)	7 (5.4%)	14 (4.8%)	0.785
LP	55 (12.9%)	16 (12.3%)	39 (13.3%)	0.787
HM	72 (16.9%)	20 (15.4%)	52 (17.7%)	0.560
CF	100 (23.6%)	30 (23.1%)	70 (23.8%)	0.870
20/400 or better	166 (39.2%)	49 (37.7%)	117 (39.8%)	0.682
Not evaluated	10 (2.5%)	8 (6.2%)	2 (0.7%)	**0.002**
Cataract with intact anterior capsule	111 (26.2%)	48 (36.9%)	63 (21.4%)	**<0.001**
Cataract with ruptured anterior capsule	82 (19.3%)	3 (2.3%)	79 (26.9%)	**<0.001**
Cataract surgery	167 (39.4%)	50 (38.5%)	117 (39.8%)	0.795

Bold indicates *p* < 0.05.

^a^Age had a non-parametric distribution, was reported as median (interquartile range, IQR) and analyzed with Mann–Whitney U Test. The Chi-square test was used to compare categorical data when expected event counts were greater than 5, and Fisher’s exact test was used when expected event counts were 5 or fewer.

^b^A sensitivity analysis performed on the entire study population, adjusting for age (years), confirmed that the protective effect of ICM for endophthalmitis after OGI remains independent of age (adjusted OR: 0.20; 95% CI: 0.04–0.86; *p* = 0.031). Furthermore, age was not significantly associated with the outcome (adjusted OR: 0.99; 95% CI: 0.94–1.03; *p* = 0.734).

*No-ICM* No intracameral moxifloxacin, *ICM* Intracameral moxifloxacin, *NLP* No light perception, *LP* light perception, *HM* hand movements, *CF* count fingers.

IOFB was found significantly more often in ICM (29, 9.9%) than No-ICM (4, 3.1%) group (*p* = 0.016). Traumatic cataract with an intact anterior capsule was observed more often in No-ICM (48, 36.9%) than ICM (63, 21.4%) group (*p* < 0.001), whereas cataracts with ruptured anterior capsule were observed more often in ICM (79, 26.9%) than No-ICM (3, 2.3%) group (*p* < 0.001). The percentage of cataract surgery during OGI repair was similar between groups (39.8% and 38.5%, ICM and No-ICM group, respectively, *p* = 0.795). Similar preoperative and postoperative visual acuity was observed in both groups with no significant difference between visual acuity scores. Preoperative and postoperative visual acuity could not be evaluated in 16 (3.8%) and 10 (2.5%) patients, respectively, due to their young age or lack of cooperation. During the pandemic preoperative COVID tests were required, resulting in a significant increase of accident-repair interval time in ICM group (*p* < 0.001) (Table 1).

Post-traumatic endophthalmitis was significantly lower in the ICM group, with only 2 of 294 patients (0.7%) developing endophthalmitis, compared with 5 of 130 (3.9%) in the No-ICM group, yielding a significant difference (*p* = 0.031) (Table 1). The group of patients under 18 years of age was analysed independently, and although a trend towards a lower rate of endophthalmitis was found in the ICM group than the non-ICM group (2/97, 2.1% vs. 2/50, 4.0%), this was not statistically significant (*p* = 0.242), probably because a larger sample size of underage patients is required. A sensitivity analysis performed on the entire study population, adjusting for age (years), confirmed that the protective effect of ICM for endophthalmitis after OGI remains independent of age (adjusted OR: 0.20; 95% CI: 0.04–0.86; *p* = 0.031). Furthermore, age was not significantly associated with the outcome (adjusted OR: 0.99; 95% CI: 0.94–1.03; *p* = 0.734) (Table 1). The relative risk of post-traumatic endophthalmitis in the ICM group compared with the no-ICM group was 0.18 (0.04–0.78), representing a relative risk reduction of 82%. The absolute risk reduction was 3.2%, corresponding to a number needed to treat of 32 to prevent one case of endophthalmitis following OGI repair (Table 2).

**Table 2:** Endophthalmitis prevention with intracameral moxifloxacin (ICM) after open globe injury (OGI) repair.

	Endophthalmitis after OGI repair
**No – ICM**	5 / 130 (3.9 %)
**ICM**	2/294 (0.7 %)
**Relative risk % (95% CI)**	0.18 % (0.04 – 0.78)
**Absolute risk reduction % (95% CI)**	3.17 % (0.84 – 3.69)
**Number needed to treat (95% CI)**	32 (28 – 119)
***P*** =	0.035

Statistics: Miettinen-Nurminen (M–N) method in epiR package.

All cases with endophthalmitis were male, had an injury with a metallic object more frequently (5; 71.4%) and had the cornea in zone 1 more frequently involved (6; 85.7%). Cultures were taken from all cases during the OGI repair; however, in the endophthalmitis cases of No-ICM group, microbiological results were not available in the case files. It is important to note that of the two cases of endophthalmitis presented in the ICM group, one was caused by a fungus (*Aspergillus spp.)* against which antibiotics are ineffective. The description of the endophthalmitis cases is presented in Table 3.

**Table 3:** Endophthalmitis cases after open globe injury repair.

Characteristics	Case 1	Case 2	Case 3	Case 4	Case 5	Case 6	Case 7
Prophylactic antibiotic intervention: ICM vs. No-ICM	No-ICM	No-ICM	No-ICM	No-ICM	No-ICM	ICM	ICM
Year of OGI	2002	2007	2010	2011	2012	2016	2019
Sex	Male	Male	Male	Male	Male	Male	Male
Age	14	1	27	43	47	4	4
Type 2 diabetes	No	No	No	No	No	No	No
Hypertension	No	No	No	No	No	No	No
Pre-operative visual acuity	HM	FFO	LP	LP	CF	FFO	FFO
Ocular trauma score grade	2	3	2	2	2	3	3
Mechanism of injury	Branch	Metallic object	Metallic object	Metallic object	Glass	Metallic object	Metallic object
Accident-repair interval (hours)	<24	<24	<24	<24	<24	<24	24–72
Culture	No data	No data	No data	No data	No data	Negative	Positive, Aspergillus
Localization	Corneal	Corneal	Corneal	Scleral	Corneal	Corneal	Corneal
Zone	1	1	1	1 and 2	1	1	1
Intraocular foreign body	No	No	No	No	No	No	No
Transoperative steroid	No	Yes	Yes	Yes	Yes	No	No
Post-operative visual acuity at week 2	HM	FFO	LP	HM	CF	LP	FFO
Traumatic cataract with intact anterior capsule	No	No	No	No	Yes	Yes	No
Traumatic cataract with ruptured anterior capsule	No	No	No	No	No	No	No
Cataract surgery	No	No	No	No	Yes	Yes	No

*ICM* Intracameral moxifloxacin, *OGI* Open globe injury, *LP* light perception, *HM* hand movements, *CF* count fingers, *FFO* fixate and follows objects.

## Discussion

This study provides strong evidence that ICM is effective in reducing the incidence of post-traumatic endophthalmitis after OGI repair, showing that endophthalmitis incidence was significantly lower in ICM (0.7%) than in No-ICM (3.8%) group (*p* = 0.031). The measures of associations of RR, ARR and NNT, further support the clinical significance of the current study findings. ICM achieved an 82% relative risk reduction and a 3.2% absolute risk reduction, corresponding to a number needed to treat of 32 patients to prevent one case of endophthalmitis. Sensitivity analysis adjusted for age confirmed that ICM remains effective at reducing endophthalmitis after OGI repair, irrespective of age. This study provides evidence supporting the effectiveness of ICM at reducing the rate of endophthalmitis in a real-world OGI repair setting.

Post-traumatic endophthalmitis remains one of the most devastating complications following OGI repair and is typically associated with worse visual acuity outcomes compared with postoperative endophthalmitis due to a variety of factors, including associated comorbidities, more virulent organisms, and possible delayed diagnosis and initiation of treatment [25]. There is a higher reported rate of infection after OGI (1/100) when compared with elective intraocular surgery (1/1000) [25]. The rate of post-traumatic endophthalmitis after a repaired OGI varies among authors [26, 27]. Andreoli et al. reported a rate of 0.9% in 558 patients [27]. Verbraeken et al. identified a 4% rate among 615 patients [28]. Ariyasu et al. found a 7.2% rate in 670 patients [29]. Essex et al. noted a 6.8% rate in 250 patients [30]. None of the previous papers report the use of prophylactic intracameral antibiotic. The use of intracameral antibiotics at the end of OGI repair has been less studied than after cataract surgery; however, its benefit has been reported in papers such as Thevi et al.'s systematic review, which includes 3 clinical trials with intravitreal or intracameral gentamicin, ceftazidime, or vancomycin. They reported that overall, additions of intravitreal or intracameral antibiotics were noted to significantly reduce the occurrence of endophthalmitis (relative risk [RR] 0.19, 95% confidence interval [CI] 0.06–0.57) and suggest that intraocular antibiotics reduce the risk of endophthalmitis after OGI repair. However, this review does not report clinical outcomes of ICM prophylaxis in OGI repair [21]. Our study found that ICM achieved an 82% relative risk reduction and a 3.2% absolute risk reduction, that is a similar finding and in agreement with Thevi systematic review [21]. Notably, there is a gap in the literature on the use of ICM after OGI repair for endophthalmitis prevention. The current study findings fill that knowledge gap and support the usefulness of ICM prophylaxis at the end of OGI repair to reduce the incidence of endophthalmitis, and to our knowledge, this is the first study to confirm the utility of ICM to reduce endophthalmitis rate after the repair of OGI. There is a large variation in the practice patterns of antibiotic prophylaxis for endophthalmitis across hospitals [5, 9, 12, 16, 31]. In our university clinic, it has been standard practice to use ICM at the end of OGI repair since 2013, and we have noticed a reduction in endophthalmitis while also improving safety since ICM has the advantage of no risk or dilution errors, and the ease of use without the need to dilute, it is readily available.

We have learned from endophthalmitis prophylaxis strategies in cataract surgeries that the use of intracameral antibiotics is very helpful in reducing the incidence. Haripriya et al. compared the postoperative endophthalmitis rate before and after initiation of ICM prophylaxis of all cataract surgeries (a total of 617,453 cataract surgeries) performed at the Aravind eye hospitals in India, and there was a significant reduction in the endophthalmitis rate, from 0.07% (214/302,815) to 0.02% (64/314,638) (*P* < 0.001) with ICM prophylaxis [13]. Bowen et al. conducted a systematic review and meta-analysis of the safety and efficacy of intracameral cefuroxime, moxifloxacin, and vancomycin at the end of cataract surgery, the average postoperative endophthalmitis incidence rates with intracameral cefuroxime, moxifloxacin and vancomycin were 0.0332%, 0.0153%, 0.0106%, respectively [5]. Rathi et al. conducted a prospective study to compare the efficacies of intracameral cefuroxime and moxifloxacin prophylaxis in reducing the incidence of acute endophthalmitis after cataract surgery in rural India, the reduction in incidence was statistically significant (*p* < 0.001) [14].

Strengths of the study include its clearly defined research question and large sample size encompassing participants of a wide range of ages and sexes, and in a real-world setting. As previously mentioned to our knowledge this is the first study that evaluate the utility of ICM to reduce the rate of endophthalmitis after OGI repair. Also, both study groups exhibit substantial demographic similarity, predominantly comprising male patients with comparable incidences of comorbidities. Despite these similarities, the group that received ICM prophylaxis at the end of surgery demonstrated a statistically significant reduction in the incidence of endophthalmitis. Remarkably, this beneficial effect of ICM remained even when encountering a significantly higher number of patients exhibiting recognised risk factors for endophthalmitis, such as delayed surgical repair beyond 24 h post-OGI, the presence of an IOFB, and a cataract with lens capsular rupture [13, 15, 25, 30, 32–34].

The study has certain limitations, including limited information on some variables from the No-ICM group, as expected in a retrospective analysis where occasional data was missing. The Ocular Trauma Score (OTS) has demonstrated prognostic value for visual acuity recovery after OGI, it has been previously studied within our institution, and published in a multivariate analysis where a lower OTS [OR = 1.09 (1.04–1.14), *p* < 0.0001) retained its statistical significance as risk factor for SVI (severe visual impairment) [35]. In the current study, the OTS documentation was incomplete in the retrospective portion of the present dataset, as a result, an unbiased OTS outcome correlation could not be performed. In cases of cataract, it was not recorded whether an intraocular lens (IOL) was implanted or not, and we do not have information on how many cases underwent pars plana vitrectomy or the type of tamponade used. Unfortunately, the microbiological report of the endophthalmitis cases of the No-ICM group was not available in the case files. No follow-up on endophthalmitis cases was analysed to assess outcomes and postoperative management. That is because the focus of this study was to demonstrate the effectiveness of moxifloxacin in preventing endophthalmitis rather than exploring the outcome after endophthalmitis.

In conclusion, this study confirms that the incidence of postoperative endophthalmitis following repaired OGI is reduced using ICM at the end of the surgical repair of OGI. Intraoperative prophylaxis following a repaired OGI should be considered a standard of care, as is the case with cataract surgery.

## Summary

### What was known before

- Traumatic open globe injuries (OGI) frequently cause severe vision loss.
- Bacterial endophthalmitis following surgical repair can significantly worsen visual outcomes in these cases.
- There is limited evidence regarding the use of intracameral moxifloxacin as prophylaxis for endophthalmitis after repaired OGI.

### What this study adds

- The incidence of postoperative endophthalmitis following repaired open globe injury is reduced with ICM administered at the end of repaired OGI.
- In a similar way to cataract surgery, intraoperative prophylaxis following a repaired open globe injury should be considered a standard of care.

## Supplementary information

Supplemental Material 1 - REPORT OF POST-SURGICAL ENDOPHTHALMITIS

## Data Availability

The datasets generated during and/or analysed during the current study are available from the corresponding author on reasonable request.
