# Supplementary material for: Incidence of post-traumatic endophthalmitis following repaired open globe injury: impact of prophylactic intracameral moxifloxacin
Source: Eye (Lond). 2026 Feb 26;40(7):1030–6. doi: 10.1038/s41433-026-04338-y (PMC13161291; doi:10.1038/s41433-026-04338-y)
Supplement: Supplementary file 1 — Supplemental Material 1 - REPORT OF POST-SURGICAL ENDOPHTHALMITIS [file 41433_2026_4338_MOESM1_ESM.doc]

**REPORT OF POST-SURGICAL ENDOPHTHALMITIS**

**REPORTE DE ENDOFTALMITIS POSTQUIRÚRGICA**

Nombre del Paciente (AP, AM, N): _________________________________________________

Teléfonos: ______________________________ Convenio: __________________________

Fecha de Diagnóstico: _____/_____/_____Quirófano:___________ Registro: ______________

Doctor 1: _____________________________________________________________________

Doctor 2: _____________________________________________________________________

Doctor 3: _____________________________________________________________________

Asistente 1: ___________________________________________________________________

Profesor responsable de la cirugía:_________________________________________________

Clínica: ______________________________________________ **OJO OPERADO:** OD / OS

**Cirugía ocular previa (en el ojo con endoftalmitis):**

| **Nombre del procedimiento** | **Lugar donde se realizo:** | **Fecha** |
| --- | --- | --- |
|  | 🞏 Oftalmo HU  🞏 Otro lugar: **_____________** |  |
|  | 🞏 Oftalmo HU  🞏 Otro lugar: **_____________** |  |
|  | 🞏 Oftalmo HU  🞏 Otro lugar: **_____________** |  |

**FACTORES PREOPERATORIOS:**

**Agudeza visual preoperatoria:**  **OD:** AVSC **OS:** AVSC

MAVC MAVC

**Motivo de consulta original, por el que el paciente acudió a su visita donde se le recomendó el procedimiento o cirugía al que se asocia la endoftalmitis: _____________________________________________________________________**

**Enfermedad médica predisponente:**

SI🞏 NO🞏 Diabetes Mellitus

SI🞏 NO🞏 Enfermedad crónica renal

SI🞏 NO🞏 Enfermedades autoinmunes (colágeno-artropatías)

🞏 Otra, especifique ____________________

🞏 Ninguna

**Infección Ocular predisponente:**

SI🞏 NO🞏 Chalazión / Orzuelo

SI🞏 NO🞏 Blefaritis

SI🞏 NO🞏 Obstrucción del conducto nasolagrimal

SI🞏 NO🞏 Conjuntivitis

🞏 Otra, especifique ____________________

🞏 Ninguno

**Factores anatómicos predisponentes:**

SI🞏 NO🞏 Pequeña abertura palpebral

SI🞏 NO🞏 Ojo hundido (Orbita profunda)

🞏 Otra, especifique ____________________

🞏 Ninguno

**Enfermedad ocular previa:**

SI🞏 NO🞏 Glaucoma

SI🞏 NO🞏 Retinopatía Diabética

SI🞏 NO🞏 Queratopatía Bulosa

🞏 Otra, especifique ____________________

🞏 Ninguno

**Corticoesteroides previos / Medicación de inmunosupresores antes de la cirugía (tópico o sistémico):**

SI🞏 NO🞏 especifique todas_______________________________

1. **FACTORES OPERATORIOS:**

**Tipo de cirugía:**

SI🞏 NO🞏 EECC

SI🞏 NO🞏 FACO

SI🞏 NO🞏 Inyección intravítrea, especifique __________________________________

SI🞏 NO🞏 Cirugía de Glaucoma, especifique__________________________________

SI🞏 NO🞏 Cirugía de Vítreo, especifique_____________________________________

SI🞏 NO🞏 Trasplante de córnea, especifique_________________________________

SI🞏 NO🞏 Reparación de herida ocular, especifique___________________________

🞏 Otras, especifique______________________________________________

**Fecha de la Cirugía:** ____/____/____ **Quirófano: 1 2 3 4 5 6**

Duración de la cirugía: ____min. Número de cirugía en ese quirófano ese día: ________

Yodo povidona tópico usado antes de la cirugía: SI🞏 NO🞏

**Anestesia**:

SI🞏 NO🞏 Sedación

SI🞏 NO🞏 Bloqueo retrobulbar o peribulbar

SI🞏 NO🞏 Intracameral

SI🞏 NO🞏 Tópica (Gel)

SI🞏 NO🞏 Tópica (Gotas)

SI🞏 NO🞏 General Balanceada

**Tipo de incisión:**

SI🞏 NO🞏 Límbica

SI🞏 NO🞏 Corneal clara

SI🞏 NO🞏 Túnel escleral

SI🞏 NO🞏 Inyección intravítrea vía pars plana

🞏 Otro, especifique______________________________________________

**Sitio de la incisión principal:**

1. Superior, indique, M___________
2. Temporal, indique, M__________
3. Otros, especifique_____________________________________________

**Tamaño de la incisión:** ________________________mm

**Medicamentos trans operatorios**

SI🞏 NO🞏 Adrenalina Intracameral

SI🞏 NO🞏 Miótico pupilar (Ej. Iloc)

SI🞏 NO🞏

**LIO implantado:**

SI🞏 NO🞏 Acrílico plegable (1 pieza)

SI🞏 NO🞏 Silicón plegable (1 pieza)

SI🞏 NO🞏 PMMA con haptica de acrilico (3 piezas)

SI🞏 NO🞏 PMMA 1 pieza (cámara posterior)

SI🞏 NO🞏 Cámara anterior

🞏 Ninguno

🞏 N / A

*Calcomanía de LIO*

**Calcomanía de LIO implantado**

**Suturas:**

1. Ninguna 2) Si Número: ___________

**Complicaciones intraoperatorias:**

SI🞏 NO🞏 Ruptura de la cápsula posterior

SI🞏 NO🞏 Pérdida de vítreo

SI🞏 NO🞏 Dislocación del núcleo en vítreo

SI🞏 NO🞏 Ampliación de herida corneal

SI🞏 NO🞏 Conversión a EECC

SI🞏 NO🞏 Todo lo de arriba

🞏 Otras:____________________________________________________

🞏 Ninguna

1. **EL DÍA DEL DIAGNÓSTICO DE ENDOFTALMITIS**

Fecha de Diagnóstico clínico de endoftalmitis: _____/_____/_____

Número de días Post Quirúrgicos al momento del Diagnóstico Clínico: ________

Inicio de los síntomas después de la cirugía: ____________________(días / semanas)

Número de días desde la aparición de los síntomas a la presentación: ___________(días)

AVSC______________________________ MAVC o PH_____________________________

PIO ______ Tratamiento para HTIO: SI🞏 NO🞏 Mencione___________________________

Escala de dolor: 0 1 2 3 4 5 6 7 8 9 10 (Leve: 1 – 3 Moderado: 4-7 Severo: 8-10)

**Síntomas:** (Indique todas las que aplique)

SI🞏 NO🞏 Dolor ocular

SI🞏 NO🞏 Disminución repentina de la visión

SI🞏 NO🞏 Blefaroedema

SI🞏 NO🞏 Hiperemia

🞏 Otros, especifique______________________________________

**Presentación clínica:**

| **Segmento Anterior y anexos:**  SI🞏 NO🞏 Córnea opaca  SI🞏 NO🞏 Celularidad (cruces): 0+ trazas 1+ 2+ 3+ 4+  SI🞏 NO🞏 Fibrina en cámara anterior 0+ trazas 1+ 2+ 3+ 4+  SI🞏 NO🞏 Flare  SI🞏 NO🞏 Hipopión  SI🞏 NO🞏 Turbidez del Vítreo  SI🞏 NO🞏 Infección de la herida  SI🞏 NO🞏 Fuga de la herida  SI🞏 NO🞏 Prolapso de Iris (exposición de úvea)  SI🞏 NO🞏 Queratitis  SI🞏 NO🞏 Hiperemia conjuntival  SI🞏 NO🞏 Quemosis | SI🞏 NO🞏 Secreción  SI🞏 NO🞏 Blefaroedema  SI🞏 NO🞏 Celulitis Orbitaria o Preseptal  🞏 Otros, especifique______________________________________  **Fondo de ojo:**  SI🞏 NO🞏 Vitritis (Vítreo Turbio)  SI🞏 NO🞏 No valorable 🞏 Palidez o Necrosis de Retina  SI🞏 NO🞏 Reflejo rojo  SI🞏 NO🞏 Reflejo blanco  🞏 Otros, especifique______________________________________ |
| --- | --- |

**Historial de aumento de la PIO después de la operación:**

1. No 2) Si Día Post-Qx_______ mm Hg________

**Cumplimiento postoperatorio del paciente a los antibióticos tópicos profilácticos:**

1. Excelente 2) Bueno 3) Regular 4) Malo

**Fotos de segmento anterior** : SI🞏 NO🞏 Fecha: _____/_______/________

1. **IMPRESIÓN DIAGNÓSTICA**

Endoftalmitis post-quirúrgica aséptica 🞏

Endoftalmitis post-quirúrgica infecciosa 🞏

Otra: 🞏 ________________________________________________________

1. **ESTUDIOS DIAGNÓSTICOS**

| **Estudios diagnósticos** | **Si** | **No** | **Fecha** |
| --- | --- | --- | --- |
| 1. Gram y Cultivo de muestra conjuntival |  |  |  |
| 1. Gram y Cultivo de raspado corneal |  |  |  |
| 1. Gram y Cultivo de humor acuoso |  |  |  |
| 1. Gram y Cultivo de vítreo |  |  |  |

**Resultado de Gram:**

| **No Microorganismos** | **Gram Positivo** | **Gram Negativo** | **Hifas** | **Levaduras** | **Muestra Insuficiente** | **Otros:** |
| --- | --- | --- | --- | --- | --- | --- |

| **Resultados** | **Cultivo** | | | **Sensibilidad** |
| --- | --- | --- | --- | --- |
|  | **+ / -** | **NA** | **Agente** |  |
| 1. Cultivo de muestra conjuntival |  |  |  |  |
| 1. Cultivo de raspado corneal |  |  |  |  |
| 1. Cultivo de humor acuoso |  |  |  |  |
| 1. Cultivo de vítreo |  |  |  |  |

**V. TRATAM**IENTO INICIAL

| **Tratamiento Menciona todos los antibióticos** | | | | | |
| --- | --- | --- | --- | --- | --- |
| Antibióticos intrevítreos inyectados | SI🞏 NO🞏 | Fecha | Nombre | Volumen | Concentración |
|  |  |  |  |
| Antibióticos en cámara anterior inyectados | SI🞏 NO🞏 |  |  |  |  |
| Antibióticos subconjuntivales | SI🞏 NO🞏 |  |  |  |  |
| Antibióticos tópicos | SI🞏 NO🞏 |  |  |  |  |

| **Terapia adyuvante con esteroides Menciona todos los esteroides** | | | | | |
| --- | --- | --- | --- | --- | --- |
| Oral | SI🞏 NO🞏 | Fecha | Nombre | Volumen | Concentración |
|  |  |  |  |
| Subconjuntival | SI🞏 NO🞏 |  |  |  |  |
| Tópico | SI🞏 NO🞏 |  |  |  |  |
| Intravítrea | SI🞏 NO🞏 |  |  |  |  |
| Ninguno 🞏 |  |  |  |  |  |

| Vitrectomía | SI🞏 NO🞏 | Fecha: | Días después del Dx. | AV al momento antes de la Cx. |
| --- | --- | --- | --- | --- |
| Lavado de Cámara Anterior | SI🞏 NO🞏 | Fecha: | Días después del Dx. | AV al momento antes de la Cx. |

1. **RESULTADO VISUAL Y EVOLUCIÓN**

**1 DÍA POST-OPERATORIO (De la Cirugía que produjo la endoftalmitis)**

Fecha:_____/_____/_____ AVSC_______ PIN HOLE_______

Hiperemia Si 🞎 No🞎 Quemosis Si 🞎 No🞎 Células: 0 1 2 3 4 Flare:____ Fibrina Si 🞎 No🞎 Hipopion Si 🞎 No🞎

Sinequias Posteriores Si 🞎 No🞎 Sinequias Anteriores Si 🞎 No🞎 Edema Corneal 0 1 2 3 4

Vitritis Si 🞎 No🞎 0 1 2 3 4 Hemovitreo Si 🞎 No🞎 Blefaroedema Si 🞎 No🞎 Dolor Si 🞎 No🞎

Claridad de medios: Si 🞎 No🞎 razones______________________________________

**3 DÍA POST-OPERATORIO (De la Cirugía que produjo la endoftalmitis)**

Fecha:_____/_____/_____ AVSC_______ PIN HOLE_______

Hiperemia Si 🞎 No🞎 Quemosis Si 🞎 No🞎 Células: 0 1 2 3 4 Flare:____ Fibrina Si 🞎 No🞎 Hipopion Si 🞎 No🞎

Sinequias Posteriores Si 🞎 No🞎 Sinequias Anteriores Si 🞎 No🞎 Edema Corneal 0 1 2 3 4

Vitritis Si 🞎 No🞎 0 1 2 3 4 Hemovitreo Si 🞎 No🞎 Blefaroedema Si 🞎 No🞎 Dolor Si 🞎 No🞎

Claridad de medios: Si 🞎 No🞎 razones______________________________________

**5 DÍA POST-OPERATORIO (De la Cirugía que produjo la endoftalmitis)**

Fecha:_____/_____/_____ AVSC_______ PIN HOLE_______

Hiperemia Si 🞎 No🞎 Quemosis Si 🞎 No🞎 Células: 0 1 2 3 4 Flare:____ Fibrina Si 🞎 No🞎 Hipopion Si 🞎 No🞎

Sinequias Posteriores Si 🞎 No🞎 Sinequias Anteriores Si 🞎 No🞎 Edema Corneal 0 1 2 3 4

Vitritis Si 🞎 No🞎 0 1 2 3 4 Hemovitreo Si 🞎 No🞎 Blefaroedema Si 🞎 No🞎 Dolor Si 🞎 No🞎

Claridad de medios: Si 🞎 No🞎 razones______________________________________

**1 SEMANA POST-DIAGNÓSTICO**

Fecha:_____/_____/_____ AVSC_______ PIN HOLE_______

Hiperemia Si 🞎 No🞎 Quemosis Si 🞎 No🞎 Células: 0 1 2 3 4 Flare:____ Fibrina Si 🞎 No🞎 Hipopion Si 🞎 No🞎

Sinequias Posteriores Si 🞎 No🞎 Sinequias Anteriores Si 🞎 No🞎 Edema Corneal 0 1 2 3 4

Vitritis Si 🞎 No🞎 0 1 2 3 4 Hemovitreo Si 🞎 No🞎 Blefaroedema Si 🞎 No🞎 Dolor Si 🞎 No🞎

Claridad de medios: Si 🞎 No🞎 razones______________________________________

Celulitis orbitaria: Si 🞎 No🞎 Ptisis bulbi : Si 🞎 No🞎 Evisceración : Si 🞎 No🞎 Enucleación : Si 🞎 No🞎

Procedimientos adicionales en este periodo SI 🞏 NO 🞏 (Poner fecha de cada procedimiento)

__________________________________________________________________________________________________

__________________________________________________________________________________________________

Diagnósticos nuevos en este periodo SI 🞏 NO 🞏

__________________________________________________________________________________________________

Tratamiento actual tópico y sistémico

__________________________________________________________________________________________________

**1 MES POST-DIAGNÓSTICO**

Fecha:_____/_____/_____ AVSC_______ PIN HOLE_______

Hiperemia Si 🞎 No🞎 Quemosis Si 🞎 No🞎 Células: 0 1 2 3 4 Flare:____ Fibrina Si 🞎 No🞎 Hipopion Si 🞎 No🞎

Sinequias Posteriores Si 🞎 No🞎 Sinequias Anteriores Si 🞎 No🞎 Edema Corneal 0 1 2 3 4

Vitritis Si 🞎 No🞎 0 1 2 3 4 Hemovitreo Si 🞎 No🞎 Blefaroedema Si 🞎 No🞎 Dolor Si 🞎 No🞎

Claridad de medios: Si 🞎 No🞎 razones______________________________________

Celulitis orbitaria: Si 🞎 No🞎 Ptisis bulbi : Si 🞎 No🞎 Evisceración : Si 🞎 No🞎 Enucleación : Si 🞎 No🞎

Procedimientos adicionales en este periodo SI 🞏 NO 🞏 (Poner fecha de cada procedimiento)

__________________________________________________________________________________________________

__________________________________________________________________________________________________

Diagnósticos nuevos en este periodo SI 🞏 NO 🞏

__________________________________________________________________________________________________

Tratamiento actual tópico y sistémico

__________________________________________________________________________________________________

| **INFORMACIÓN** | **NOMBRE** | **FIRMA** |
| --- | --- | --- |
| Residente que llena reporte inicial |  |  |
| Profesor Responsable de Cirugía |  |  |
| Profesor de Clínica de Retina |  |  |
